# Investigating the NF-κB signaling pathway in heart failure: Exploring potential therapeutic approaches

**DOI:** 10.1016/j.heliyon.2024.e40812

**Published:** 2024-11-30

**Authors:** Mohsen Ghiasi

**Affiliations:** Rajaie Cardiovascular Medical and Research Center, Iran University of Medical Sciences, Tehran, Iran

**Keywords:** Heart failure, NF-κB pathway, Cell signaling, Inflammation

## Abstract

Heart failure (HF) syndrome is of great interest as an emerging epidemic. Due to the increasing elderly population worldwide, the total number of HF patients is increasing every day. This disease places a significant economic burden on the healthcare and treatment systems of developing societies, and this situation is very concerning. Despite many advances in the diagnosis and treatment of cardiovascular diseases, HF is still the main cause of death worldwide. This clinical syndrome has many cellular and molecular complications, which are often aggravated by increased levels of pro-inflammatory cytokines, which lead to adverse clinical outcomes. Nuclear factor kappa B (NF-κB), a pivotal family of transcription factors, plays a crucial role in various biological processes, particularly in inflammation, immune response, cell proliferation, and cell survival. Studies show that the NF-κB signaling pathway plays a role in modulating cardiac regeneration, apoptosis, and myocardial fibrosis. It has been found that the NF-κB signaling pathway can affect heart function and HF through the regulation of matrix metalloproteinases and fibrotic mediators. Also, the NF-κB pathway regulates cell activities in cardiac cardiomyocytes and regulates the function of this organ by establishing a precise interaction between apoptosis and pyroptosis. However, the exact molecular mechanisms of this influence have not been well defined and there are many scientific gaps in this matter. This review tries to highlights potential therapeutic strategies to target NF-κB, including the use of anti-inflammatory agents and genetic modulation, which may provide new ways to reduce cardiac fibrosis and improve outcomes in HF patients. Certainly, increasing understanding of the multifaceted role of NF-κB in HF can lead to innovative treatments aimed at reducing the growing number of patients worldwide.

## 1 Introduction

Heart failure (HF) affects more than 26 million people worldwide and is considered an important challenge in global health [1]. According to statistics, approximately 6.7 million Americans age 20 and older have heart failure. It is expected that this number will reach 8.5 million people by 2030 [2]. HF is so important that HF syndrome was first described about 25 years ago as an emerging epidemic [3]. Also, this disease imposes a great economic burden on the global healthcare system. The economic impact of HF is significant, with healthcare costs constituting a major component of this impact. In addition, HF also has indirect costs on the economy of countries, because it reduces the productivity of human resources [4,5]. For example, an assessment in Russia found that HF leads to an annual economic impact of approximately RUB 81,86 billion (about 1.1 billion USD) in that country [6]. This disease is a complex clinical syndrome in which sufferers face structural and functional disorders of the heart [7,8]. Research has proven that HF patients show increased levels of pro-inflammatory cytokines, which causes adverse clinical outcomes in them [9,10]. Several studies show that there is a strong connection between the activation of the NF-κB pathway and important diseases such as Alzheimer's [11], cancers [12], and cardiovascular diseases, including HF [13]. For example in a study shown that patients who received left ventricular assist devices showed improved heart function along with reduced activity of cardiac NF-κB [14]. Nuclear factor kappa B (NF-κB) is a family of transcription factors that play a very important role in biological processes. This family is very important in the inflammation pathway, immune response, cell proliferation, differentiation and, cell survival [15,16]. Different stimuli involved in the development of HF, including reactive oxygen species (ROS), hypoxia, and inflammatory cytokines, activate the NF-κB pathway [17]. Inflammation is a natural body mechanism and a fundamental strategy for resistance to pathogens and tissue repair [18]. But if inflammation becomes chronic, it can cause serious damage to the body, myocardial infarction, high blood pressure, and atherosclerosis, all of which contribute to heart failure, are caused by chronic inflammation [19,20]. Research has shown that high levels of circulating pro-inflammatory biomarkers in HF patients are associated with disease severity [21]. Due to the fact that the activation of the NF-κB pathway causes the generation and activation of inflammatory cytokines, chemokines, macrophages, neutrophils, dendritic cells, T cells and, B cells, and the subsequent inflammatory process which all lead to damage in the tissues and organs of the cardiovascular system [22], so trying to modulate the inflammatory response in these patients can be evaluated as a new strategy. Recent advances in targeted drug delivery systems, especially nanotechnology-based systems, have been promising to increase therapeutic efficacy in various diseases, including cancers and infectious diseases [[23], [24], [25]]. Therefore, the use of targeted drug delivery methods and influencing the NF-κB signaling pathway can be considered an effective and innovative approach in the treatment of HF. Today, common medical treatments for HF include ACE inhibitors, β-blockers, and diuretics, which reduce symptoms and improve patients' quality of life. But it may also have certain side effects and not all patients with HF respond positively to these drugs. Therefore, research on new and targeted treatment methods is strongly felt. Considering that NF-κB is involved in pathophysiological processes that lead to cardiac regeneration, inflammation, and apoptosis. The present review aims to address the role of NF-κB in HF, its signaling pathways, and the potential therapeutic targets of this pathway in HF.

## 2 NF-κB signaling pathways

NF-κB as an inducible transcription factor that consists of a family of five proteins in mammals was discovered about 30 years ago [26]. NF-κB pathway consists of canonical and non-canonical pathways. The activation of both NF-κB pathways is highly regulated [27]. NF-κB exists in the cell cytoplasm in an inactive form, inactive NF-κB bound to inhibitors (IκBs). If the cell is exposed to various factors such as cytokines, oxidative stress, mechanical damage, and various chemical agents, IκBs are phosphorylated and decomposed, which causes NF-κB dimers to be transferred to the nucleus. By translocating NF-κB to the nucleus, the expression of genes involved in inflammation, cell survival, and apoptosis is affected [[28], [29], [30]].

## 3 Role of NF-κB signaling in heart failure

### 3.1 Role of NF-κB in cardiac remodeling

**Fig. 1:**
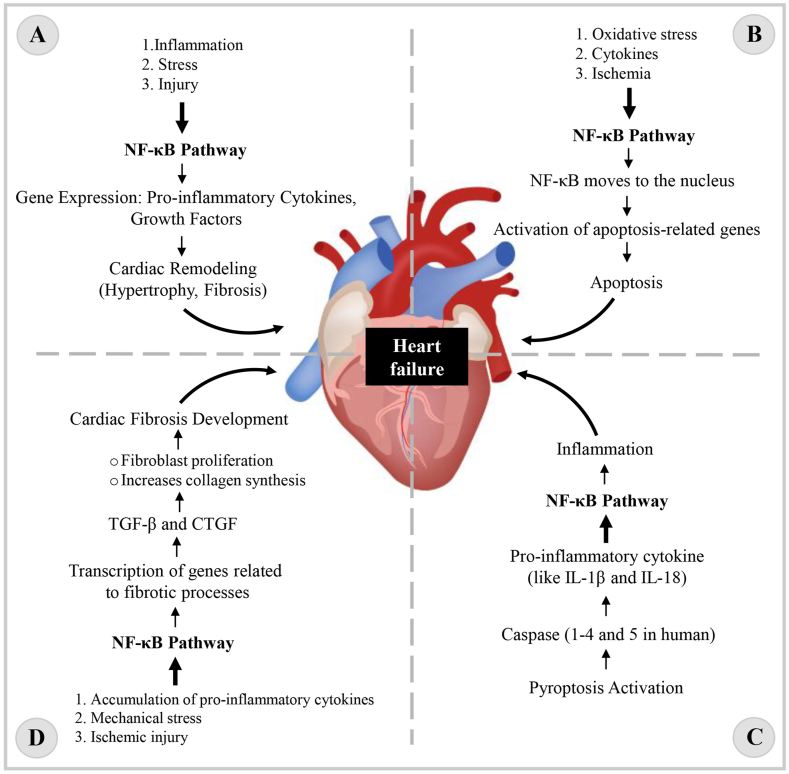
Overview of the role of the NF-κB pathway in HF. **A.** Role of NF-κB in Cardiac Remodeling, **B.** NF-κB and Apoptosis, **C.** NF-κB and Pyroptosis, **D.** NF-κB and Cardiac Fibrosis.

Cardiac remodeling is a common characteristic observed in nearly all types of heart disease [31]. Cardiac remodeling often causes a change in the thickness of the heart wall, this causes a decrease in ventricular ejection and an imbalance in the neurohumoral system, as a result of which the ventricular reconstruction is intensified and finally causes HF [[32], [33], [34]]. The heart is a complex tissue structure composed of several cell populations. In the structure of this tissue, cardiac and non-cardiomyocyte cells including fibroblasts, immune cells, etc. are observed [35]. The process of cardiac remodeling is well regulated by various mechanisms including apoptosis, proliferation, migration, and differentiation. Matrix metalloproteinases (MMPs) are a large family of enzymes involved in important biological processes such as repair, cancer and metastasis, inflammatory response, and cellular activities [36,37]. Research has shown that the expression level of MMPs increases after myocardial infarction (MI) and if the expression of the MMPs gene is disturbed, the function of the left ventricle is disturbed in heart failure patients [38]. Studies show that inhibiting the transcription factor NF-κB reduces the production of MMP1, 3, and 9 in vascular smooth muscle cells [39]. So, one of the crucial roles of NF-κB is influencing the expression of MMPs and their role in HF. The schematic Fig. 1A shows the cardiac remodeling.

### 3.2 NF-κB and apoptosis and pyroptosis

One of the important events during HF is cell death. Apoptosis, a type of active cell death, apoptosis an important role in embryonic development and the maturation of lymphoid cells by selectively removing certain cells from the body [40]. Research shows that myocyte apoptosis by itself is sufficient to induce HF in animal models [41]. NF-κB also plays a dual role in apoptosis. NF-κB plays an effective role in promoting cell survival. Evidence shows that the transcription factor NF-κB is a major factor in induced anti-apoptotic mechanisms. Studies have shown that NF-κB activates the transcription of genes that encode anti-apoptotic proteins, such as Bcl-2 and Bcl-xL, which inhibit the process of apoptosis [42,43]. NF-κB has been shown to induce the transcription of some genes involved in cell survival, including FLIP. When NF-κB is activated by blocking TNFR1, apoptosis does not occur and cells respond in other ways such as participating in inflammatory responses by producing cytokines [44]. In some conditions, NF-κB may promote apoptosis (pro-apoptosis role). Depending on the specific cell type and stimulus involved, NF-κB activation may lead to pro-apoptotic activity [45]. In general, long-term activation of NF-κB causes chronic inflammation characterized by high levels of cytokines such as TNF-α and IL-1β, which induces apoptosis and causes cardiac dysfunction [46]. Schematic Fig. 1B, shows the effect of apoptosis on the induction of HF. In summary, although NF-κB can increase cell survival by activating anti-apoptotic genes, its chronic activation during HF leads to inflammation, cytokine production, and finally apoptosis of cardiomyocytes, which play a role in the progression of HF.

Pyroptosis is a cell death process that is associated with cell swelling until membrane rupture and the release of intracellular contents, pyroptosis is closely related to the inflammatory response [47]. Unlike pyroptosis, in apoptosis the cell contents do not leak out, and cell inflammation is largely prevented [48]. The most important difference between pyroptosis and other cell deaths is the activation of a large number of inflammatory factors and the activation of pro-inflammatory signals [49]. Pyroptosis is a double-edged sword, on the one hand, pyroptosis can help cell homeostasis and prevent excessive cell proliferation, and on the other hand, excessive pyroptosis causes cardiac fibrosis, myocardial hypertrophy, and myocardial death [50]. Research has shown that the proinflammatory cytokines IL-1β and IL-18, which are involved in pyroptosis, have negative contractile effects on the heart both *in vitro* and *in vivo* [[51], [52], [53]]. Studies show that the NF-κB signaling pathway in cardiovascular diseases activates pyroptosis in cardiomyocytes [54]. Refer to schematic Fig. 1C.

### 3.3 NF-κB and cardiac fibrosis

Myocardial fibrosis is caused by the proliferation of cardiac fibroblasts, in myocardial fibrosis we see excessive deposition of extracellular matrix proteins (ECM), including collagen [55]. Almost all heart diseases involve myocardial fibrosis. Recent studies have shown that myocardial fibrosis is the main factor in the development and progression of HF [56]. Excessive myocardial fibrosis reduces cardiac compliance and ejection fraction, thereby contributing to HF [57]. Research shows that the NF-κB pathway is activated due to the accumulation of pro-inflammatory cytokines, mechanical stress, and ischemic injury, as a result of the activation of this pathway, the transcription of genes related to fibrotic processes, such as transforming growth factor beta (TGF-β) and factor Connective tissue growth factors (CTGF) are important mediators of fibrosis [58,59]. In addition to stimulating fibroblast proliferation, TGF-β also increases collagen synthesis, which contributes to the fibrotic response [60,61]. It was reported in a study that miR-26a is regulated in cardiac fibrosis through NF-κB. In this study, it was found that inhibition of NF-κB in cardiac fibroblast restores miR-26a expression and is a feedback regulatory mechanism in cardiac fibrosis [62]. In an evaluation conducted on the rat, it was found that Liquiritin (a flavonoid extract from licorice) can improve heart function, and reduce oxidative damage and inflammatory response in the rat heart. In this study, it was found that Liquiritin protects heart tissue against myocardial fibrosis following MI by inhibiting the expression of CCL5 (a pro-inflammatory chemokine) and the NF-κB pathway [63]. In general, the interaction between NF-κB signaling and the development of myocardial fibrosis in HF suggests that targeting this important pathway could lead to the creation of a new therapeutic strategy aimed at reducing cardiac fibrosis and improving cardiac function in patients with HF. The involvement of the NF-κB pathway in the process of HF through cardiac fibrosis is shown in Fig. 1D.

## 4 Regulation of the NF-κB pathway and its therapeutic role in HF

Considering the important and critical role of NF-κB in heart failure, targeting it signaling pathways could provide a potential therapeutic strategy. There are different approaches to targeting. Taking into account the fact that the expression of cytokines in HF causes chronic inflammation in heart tissues, then managing this path can be useful [64,65]. Anti-inflammatory drugs such as aspirin, sodium salicylate and, dexamethasone can suppress the activation of the NF-κB pathway. These drugs inhibit NF-κB by blocking the degradation of IκBα and thereby inhibiting NF-κB [66,67]. To regulate the non-canonical pathway of NF-κB, the interaction between transcription factors can be used. RelB/p52 heterodimers are considered as transcription factors of the non-canonical NF-κB pathway. p100 is the precursor of p52, which functions as an IκB-like protein. Therefore, p100 processing is the central event of the non-canonical NF-κB signaling pathway, which is regulated by the NIK-IKKα axis [68,69]. Therefore, by inhibiting p100, it can manage the non-canonical pathway of NF-κB. In a study using a mouse model in which HF was induced by left coronary artery occlusion, it was found that Sirtuin 1 alleviates HF-induced complications in mice by affecting the NF-κB p65/miR-155/BDNF signaling cascade. Indeed, the overexpression of Sirtuin 1 upregulates BDNF, improves cardiac function, and reduces apoptosis in cardiomyocytes [1]. In another study, it was reported that the absence of NF-κB p50 subunit improves the initial survival of HF model mice and reduces left ventricular dilatation after myocardial infarction. In this research, the content of collagen and the expression of matrix metalloproteinase-9 (MMP-9) in p50 knockout mice were significantly lower after myocardial infarction. According to these results, NF-κB may be an attractive target for the treatment of HF [17]. Another study conducted in male mice reported the role of NF-κB p65. In this study, HF was induced in mice by coronary artery occlusion. Investigation of the NF-κB p65 pathway was conducted over 4 weeks, the results showed that continuous myocyte NF-κB p65 activation in HF enhances cardiac regeneration by anti-inflammatory, pro-fibrotic and pro-apoptotic effects [70]. In a recent study, it was found that Salvianolic acid A (SAA) reduces heart failure in model mice (C57BL/6N mice) by regulating TLR/Myd88/TRAF/NF-κB and p38MAPK/CREB signaling pathways. The results of this study showed that SAA reduces the release of pro-inflammatory cytokines and mediators through NF-κB and p38 MAPK pathways [71]. Research has shown that inhibiting NF-κB can rescue heart function by restoring calcium genes in a model of Duchenne muscular dystrophy. In this study, which was carried out in model murine with dystrophic hearts (mdx), it was found that NF-κB ablation rescues cardiac function. This physiological improvement is associated with a signature of calcium-upregulated genes [72]. In summary, it can be said that regulation of the NF-κB pathway for therapeutic interventions in HF is a new approach that seems to be effective as is clear in the mentioned researches. Considering the complex role of NF-κB in mediating inflammatory responses and cardiac regeneration, this pathway is considered a special target for HF treatment. The results of these investigations show that interventional strategies including the use of anti-inflammatory agents, along with the modulation of specific transcription factors in the NF-κB signaling cascade, seem to be efficient in experimental models.

## 5 Discussion

HF is a heterogeneous clinical syndrome that accounts for significant annual mortality. In the last decade, although the incidence of HF has stabilized or decreased in high-income countries, its prevalence is increasing due to the aging population in these countries [73]. This challenge is more worrying in developing countries. For example, South Asia has a quarter of the world's population and accounts for 60 % of the global burden of heart disease. The increased risk of HF in these regions becomes more concerning when the disease appears at a younger age than in most other countries [74]. In general, the costs of caring for patients with HF, including the costs of hospitalization and chronic treatments, are very high [75]. Several classes of drugs are used to control HF in patients. Currently, clinical practice guidelines recommend that drugs such as Angiotensin-converting enzyme (ACE) inhibitors, Angiotensin receptor blockers (ACE inhibitors), Beta Blockers, and Diuretics be used. However, a very important challenge facing doctors is the simultaneous use of several drugs, achieving the target doses, and managing their side effects. Factors such as blood pressure issues, poor kidney function, hyperkalemia, and other underlying conditions in patients may prevent the achievement of appropriate doses for all drugs [76]. In general, research has shown that molecular pathways and signaling cascades can play an effective role in human diseases. For example, the NF-κB pathway is related to various diseases such as cancers, neurodegenerative diseases [77], diabetes [78], inflammatory diseases [79], autoimmune diseases [80], and cardiovascular diseases [22]. The inflammatory process in HF is one of the key events that occurs in its pathology. Studies have shown that chronic activation of NF-κB is associated with increased levels of inflammatory cytokines such as IL-1β and TNF-α. This confirms that prolonged NF-κB signaling causes chronic inflammation in the patient, which ultimately compromises heart health [59]. Therefore, it is suggested disrupting the activation of NF-κB, which leads to the transcription of multiple pro-inflammatory cytokines, including TNF-α and IL-1β, we can hope to reduce the inflammatory process in patients with HF. This approach aims to improve to improve the overall and general condition of patients and reduce their symptoms. The approach of targeting the activity of this pathway has also previously attracted attention in chronic inflammatory diseases such as Rheumatoid arthritis (RA) [81], Multiple Sclerosis (MS) [82] and, inflammatory bowel disease [83], in which NF-κB activity is persistently elevated. Of course, it should be noted that due to the very complex roles of NF-κB, excessive inhibition of this pathway can cause adverse effects and disrupt immune responses.

In addition, chronic activation of NF-κB leads to persistent inflammation in cardiac tissue, which induces apoptosis and fibrosis in cardiomyocytes. Research has shown that ROS can activate Toll-like receptor 4 (TLR4) [20]. This process subsequently activates NF-κB again, creating a feedback loop that exacerbates inflammation and fibrosis in the tissue. Additionally, oxidative stress also induces cell death and exacerbates cardiac dysfunction by impairing mitochondrial function [84]. Overall, managing oxidative stress by modulating the NF-κB pathway could be a promising therapeutic strategy to control inflammation in cardiac tissue in HF. The use of drugs such as Nonsteroidal Anti-Inflammatory Drugs (NSAIDs), glucocorticoids such as dexamethasone, proteasome inhibitors, and natural compounds can reduce ROS [27,85]. It has been well established that proteasome inhibitors prevent the degradation of IκB proteins, ultimately causing NF-kB to remain inactive in the cytoplasm and preventing the NF-κB pathway from being activated [86]. Therefore, considering this issue, it seems that the use of compounds that can control endogenous ROS in cardiac cells may be useful in managing the NF-κB pathway in patients with HF. However, further scientific and clinical studies are needed to confirm this definitively.

Despite significant advances in recent years in understanding the molecular and inflammatory mechanisms associated with HF, there is a significant lack of literature that specifically addresses the signaling pathways involved in this complex disease. This review attempted to provide a comprehensive discussion of the role of molecular signaling, particularly the NF-κB pathway, in the pathophysiology of heart failure. However, one of the limitations of this article was the insufficient scope of detailed molecular studies on this topic. This highlights the critical need for further research to elucidate the signaling mechanisms and their implications for therapeutic intervention in patients with HF. Undoubtedly, expanding our understanding of the role of the NF-κB signaling pathway in heart failure could pave the way for the development of novel therapeutic strategies aimed at improving the quality of life for these patients. Therefore, future research using animal models is essential to bridge this knowledge gap and confirm the potential of targeting these pathways to improve the clinical management of HF.

## 6 Conclusion

Considering that the NF-κB pathway is a critical mediator for the inflammatory and apoptotic processes involved in HF. So, understanding its role in the pathophysiology of HF may lead to the development of new therapeutic strategies. Given that the NF-κB pathway is a critical mediator for the inflammatory and apoptotic processes involved in HF. So, understanding its role in the pathophysiology of HF may lead to the development of new therapeutic strategies. The key role of this pathway in various diseases, including cancer, has been well studied, but there is still no detailed understanding of the role of the NF-κB pathway in heart diseases, especially HF. Certainly, in the future, with the increase of research in this field, more secrets of this pathway will be revealed.

## Funding statement

This research has not received any funding from governmental or private institutions.

## Declaration of competing interest

The authors declare that they have no known competing financial interests or personal relationships that could have appeared to influence the work reported in this paper.
